# Evolutionary and Molecular Aspects of Indian Tomato Leaf Curl Virus Coat Protein

**DOI:** 10.1155/2012/417935

**Published:** 2012-12-11

**Authors:** Sivakumar Prasanth Kumar, Saumya K. Patel, Ravi G. Kapopara, Yogesh T. Jasrai, Himanshu A. Pandya

**Affiliations:** ^1^Department of Bioinformatics, Applied Botany Center, University School of Sciences, Gujarat University, Ahmedabad 380 009, India; ^2^Department of Botany, University School of Sciences, Gujarat University, Ahmedabad 380 009, India

## Abstract

Tomato leaf curl disease (ToLCD) is manifested by yellowing of leaf lamina with upward leaf curl, leaf distortion, shrinking of the leaf surface, and stunted plant growth caused by tomato leaf curl virus (ToLCV). In the present study, using computational methods we explored the evolutionary and molecular prospects of viral coat protein derived from an isolate of Vadodara district, Gujarat (ToLCGV-[Vad]), India. We found that the amino acids in coat protein required for systemic infection, viral particle formation, and insect transmission to host cells were conserved amongst Indian strains. Phylogenetic studies on Indian ToLCV coat proteins showed evolutionary compatibility with other viral taxa. Modeling of coat protein revealed a topology similar to characteristic Geminate viral particle consisting of antiparallel **β**-barrel motif with N-terminus **α**-helix. The molecular interaction of coat protein with the viral DNA required for encapsidation and nuclear shuttling was investigated through sequence- and structure-based approaches. We further emphasized the role of loops in coat protein structure as molecular recognition interface.

## 1. Introduction

Tomato leaf curl virus (ToLCV) is one of the most devastating causal agents of tomato (*Solanum lycopersicum*) crop which had emerged causing damage and encroaching new areas in tropical and subtropical continents every year. Plant-infecting geminiviruses belong to the family Geminiviridae in which *Begomovirus* is one among the genera possessing both mono- and bipartite genomes that infect especially dicotyledonous plant species [1]. The disease is marked by symptoms such as yellowing of leaf lamina with upward leaf curl as well as distortion, reduction in internodes, new leaves size reduction, wrinkle facade, stunted growth, and dissemination of flower from plant before onset of fruiting. ToLCV is primarily transmitted by sweet potato whitefly (*Bemisia tabaci*) and silver leaf whitefly (also called Biotype B; *Bemisia argentifolii*). Whiteflies harboring virus can nonspecifically infect a wide spectrum of plant crops and weeds including eggplant, potato, tobacco, pepper, and common bean. Infected plants seem healthy but develop symptoms leading to enormous economic loss [2].

In Indian subcontinent, ToLCV is a major problem for tomato-growing regions as several reports on new strains have been documented including New Delhi, Lucknow, Bangalore, Varanasi, Mirzapur, Vadodara, and so forth and posed a threat to crop productivity [6]. Indian ToLCV isolates are mostly monopartite (DNA-A) in nature with few isolates possessing bipartite (DNA-A and DNA-B) genome organization such as tomato leaf curl New Delhi virus (ToLCNDV) and tomato leaf curl Palampur virus (ToLCPalV) [7]. Both DNA-A and DNA-B are single-stranded (ss) DNA genomes of approximately 2.7 kb size and encode viral factors essential for viral replication, encapsidation, transmission, and systemic spread [8]. Jyothsna et al. 2012 reported tomato leaf curl Gujarat virus (ToLCGV) possesses monopartite genome and is infectious expressing systemic symptoms in *Nicotiana benthamiana* and tomato [9]. An increased symptom severity and shortened incubation period required for symptom expression was noticed when ToLCGV was coinoculated with betasatellite of tomato yellow leaf curl virus Thailand (TYLCTHB) resulted in yellow mottling [9]. The molecular relationship of ToLCGV-[Vad], an isolate from Vadodara district of Gujarat, with other strains revealed that it belongs to Old World *Begomoviruses *and established a closely related cluster with other North Indian strains including ToLCGV-(Varanasi)-[Var] and ToLCGV-(Mirzapur)-[Mir] based on the DNA-A sequence alignment [10].

The measure “breeding for resistance” conceptualizes the introduction of resistance genes found in wild tomato species into tomato cultivars to develop resistance against diseases. Kunik et al. 1994 demonstrated that tomato plants transformed with TYLCV coat protein were found to be virus-resistant [11]. In India, *Agrobacterium tumefaciens* mediated transformation of coat protein gene was carried out to develop ToLCV tolerant/resistant transgenic tomato plants under glass house conditions [12]. Transgenic tomato plants containing cucumber mosaic virus coat protein gene was also successfully transformed [13]. An asymmetric synergism and virulent pseudorecombinant between ToLCNDV and ToLCGV was reported by Chakraborty et al. 2008 and found enhanced pathogenicity when tested in *N. benthamiana*,* N. tabacum,* and *S. lycopersicum *[14]. An evidence for natural recombination was observed between tomato leaf curl Bangalore virus (ToLCBV), ToLCBV [Ban 5], and ToLCBV [Kolar] and examined the possibility of recombination between strains/species that coexist within the same geographical location [15]. Hence, tremendous consideration should be given to study the biological and molecular properties of this newly emerging causal agent.

In the present study, we examined the evolutionary and molecular prospects of ToLCGV-[Vad] coat protein. Sequence analysis of coat protein revealed that amino acids essential for systemic infection, viral particle formation, and insect transmission to host cells were evolutionarily compatible when compared to non-Indian isolates giving clues of evolutionary conservativeness. Further, molecular modeling of coat protein provided a topology similar to characteristic Geminate viral particle. Electronic properties of coat protein facilitated its interaction with viral DNA with the loop element acting as molecular recognition interface which is one of the major findings of the present study.

## 2. Materials and Methods

### 2.1. Protein Sequence Retrieval

The coat protein of ToLCGV-[Vad] (accession no. AAL78666.1) was retrieved from NCBI database (http://www.ncbi.nlm.nih.gov/) [16]. Coat proteins from Indian strains (Bangalore-CAA88227.1, Bangalore (Ban4)-AAD51286.1, Bangalore (Ban5)-AAK19178.1, Bangalore (Kolar)-AAL26553.1, Varanasi-AAO25668.1, Kelloo-AAM21566.1, Karnataka-AAB08929.1, New Delhi (Mild)-AAA92817.1, and Lucknow-CAA76209.1) were also obtained for multiple sequence alignment and phylogenetic analysis.

### 2.2. Protein Family Classification

The family of coat protein was studied using a combination of programs, namely, NCBI CD- Search (http://www.ncbi.nlm.nih.gov/Structure/cdd/wrpsb.cgi, database searched: CDD v3.03–42251 PSSMs) [17], PSI-BLAST (http://blast.ncbi.nlm.nih.gov/Blast.cgi) [18], and Pfam (http://pfam.sanger.ac.uk/) [19]. CD-Search is a NCBI's interface to search Conserved Domain Database (CDD) which utilizes RPS-BLAST (Reverse-PSI-BLAST; a variant of PSI-BLAST) to scan a set of precalculated position specific scoring matrices (PSSMs) using a protein query. PSI-BLAST (position-specific-iterated BLAST) uses initial matches to query sequence to build scoring matrix and appends additional matches to the matrix by an iterative search method in order to detect remote homologs. Pfam designates protein family by HMM (Hidden Markov Model)-based search (default settings were chosen and Pfam-A significant matches were only considered) over known protein family classifiers.

### 2.3. Analysis of Nuclear Localization Signal and Its Prediction

Nuclear localization signals (NLSs) were predicted using cNLS Mapper (http://nls-mapper.iab.keio.ac.jp/) [20] as coat protein that is known to be karyophilic [21]. cNLS Mapper is a computer program that predicts NLS by activity-based profile search and an additivity-based motif scoring function in different classes of importin-*α*/*β* pathway-specific NLS. The prediction was made with a score cut-off of 5.0 and searched for both mono- and bipartite NLSs with a long linker (13–20 amino acid length) as ToLCGV possesses mono-bipartite genome organization [6]. Classic NLS typically rich in basic amino acids such as lysine and arginine, the counts of basic amino acids was performed manually in the above predicted NLSs. Comparison with literature-reported NLS specific to BR1 nuclear export family was carried out to examine the pattern of nuclear localization. This was achieved using pairwise sequence alignment using EMBOSS Stretcher (http://www.ebi.ac.uk/Tools/psa/emboss_stretcher/) [22] with a representative family protein member (BR1 nuclear shuttle protein from squash leaf curl virus(SqLCV); NCBI accession No. NP_047247.2) against the coat protein of study. Both sequences were aligned using EBLOSUM62 scoring matrix with a gap opening and extending penalty of 12 and 2.

### 2.4. Multiple Sequence Alignment and Phylogenetic Analysis of Coat Proteins

Coat protein sequences of Indian strains were used for the analysis of multiple sequence alignment (MSA). MSA was performed using EBI ClustalW program (http://www.ebi.ac.uk/Tools/msa/clustalw2/) [23] in which the sequences were aligned pairwise initially (gap open penalty = 10, gap extension penalty = 0.1, matrix = Gonnet) and then the best local pairs (gap open penalty = 10, gap extension penalty = 0.20, matrix = Gonnet) were clustered by Neighbour-joining (NJ) technique. Subsequently, an alignment file in ClustalW format was generated and specified as input to draw phylogenetic tree using Phylip version 3.68 package (http://evolution.genetics.washington.edu/phylip/) [24]. NJ algorithm was used to draw tree with inclusion of branch length.

### 2.5. Structure Modeling of Coat Protein

Sequence-based similarity searching was initially executed in NCBI nonredundant (NR) database using BLASTp program (http://blast.ncbi.nlm.nih.gov/Blast.cgi) [25] with default settings to find a close homolog with known 3D protein structure information is known. Similarly, BLAST based homolog search in RCSB Protein Data Bank (PDB; http://www.rcsb.org/pdb/home/home.do) [26] was also carried out. Both of these procedures yielded no close homologs. So, we opted to model the coat protein using homology domain modeling and remote-based homology modeling.

### 2.6. Disorderness Prediction

In order to characterize regions of sequences in coat protein which can be efficiently modeled, disorderness prediction was made. Disordered residue was identified using DISOPRED server (http://bioinf.cs.ucl.ac.uk/disopred/) [27] with a filter threshold of 5% and a false positive threshold of 2%. The disorderness is predicted by scanning the available sequence records in the PDB and then matches the electron density map to identify the missing coordinates. As a result, atomic coordinates of such amino acids will not be available for modeling of the protein and has the greater possibility of producing an irregular loop region in the modeled coat protein. Thus, manual search (only at the N-and C-terminals) for disordered sequence window in DISOPRED predictions was performed with the intention of excluding the corresponding region for modeling the coat protein. It was also ensured that disordered residue reported in the intervening sequence positions was left out so that the structure model did not possess any gaps.

### 2.7. Homology Domain Modeling

Robetta server (http://robetta.bakerlab.org/) was used for modeling the coat protein in which Ginzu, a hierarchical domain parsing and modeling protocol was adopted [28]. The input sequence (coat protein excluded with disorderness) was initially searched using BLAST, PSI-BLAST, FFAS03 (http://ffas.burnham.org/), and 3D-Jury (http://meta.bioinfo.pl/) to obtain information on homologous regions which are then modeled with their comparative modeling protocol. Unassigned (i.e., nonhomologous as identified in the first stage) regions were then parsed to model as domain linkers using a combination of approaches, namely, HMMER search (http://hmmer.janelia.org/) over Pfam-A database and an MSA (produced from initial PSI-BLAST results) based search over NCBI NR database. Subsequently, K*Sync alignment method was utilized to predict elements that are obligated to the fold to produce a single default alignment by dynamic programming. Best five models were generated after loop modeling, domain assembly, and side-chain packing.

### 2.8. Remote-Based Homology Modeling

Efforts were also carried out to predict structure of coat protein using remote-based homology modeling approach with the help of protein homology/analogy recognition engine (Phyre) version 2.0 (http://www.sbg.bio.ic.ac.uk/phyre2/) [29]. In the first step, profile was constructed using five iterations of PSI-BLAST against NR sequence database. The query secondary structure was predicted using three independent prediction programs (PSI-PRED (http://bioinf.cs.ucl.ac.uk/psipred/), SSPro (http://download.igb.uci.edu/sspro4.html) and Jnet (http://www.compbio.dundee.ac.uk/Software/JNet/jnet.html)) and a consensus prediction was made consequently. This profile and secondary structure were then scanned against the fold library using a profile-profile alignment algorithm to generate 3D models. Followed by a reconstruction procedure in the last stage, side-chains are packed using rotamer library and best models (selected based on confidence and sequence coverage) were returned.

### 2.9. Energy Minimization and Structure Validation of Models

The modeled structures were energy minimized using a utility in Tripos Benchware 3D Explorer (academic version; Tripos: A Certara company, http://www.tripos.com/) [30] with AMBER7 force field. Modeled structures were then validated for structure correctness and stereochemistry using Ramachandran plot [31] from RAMPAGE server (http://mordred.bioc.cam.ac.uk/~rapper/rampage.php) [32]. Based on the percentage of favourness and frequency of outliers, the models were selected and used for further analysis.

### 2.10. Prediction of Sequence- and Structure-Based DNA Binding Properties

*In vitro* studies showed that coat proteins from Geminiviridae family bind nonspecifically with both ss- and ds-viral DNA [21, 33, 34]. To elucidate the role of DNA binding abilities of coat protein, sequence- and structure-based approaches were used. BindN (http://bioinfo.ggc.org/bindn/) employs support vector machines (SVMs) trained from data instances such as side chain *pK*_a_ value, hydrophobicity index, and molecular mass of an amino acid [35]. A specificity of 80% with a filter threshold of 5% was chosen to avoid overwhelmed predictions. PreDs (http://pre-s.protein.osaka-u.ac.jp/preds/) makes use of molecular surface to evaluate electrostatic potential, local, and global curvatures of the PDB queried structure to predict potential dsDNA binding sites [36]. The modeled coat protein was defined as input with validation chosen from scoring functions.

### 2.11. Viral DNA Structure Modeling and Docking with Coat Protein

Canonical viral DNA was modeled using 3D-DART (3DNA-Driven DNA Analysis and Rebuilding Tool; http://haddock.chem.uu.nl/dna/dna.php) web service with default introduction of parameters for bends (roll, tilt, and twist) [37]. It uses 3DNA “fiber” module to generate canonical DNA structure and “find_pair” and “analyze” modules to produce a corresponding base pair (step) parameter file. The parameter file was used to set up local and global bends in the DNA structure file which are then remodeled finally using “rebuild” component to return PDB formatted DNA structure file. The docking phase was carried out using HADDOCK (High Ambiguity Driven biomolecular DOCKing; http://haddock.chem.uu.nl/) program [38] with the modeled coat protein and ds-viral DNA as inputs. Residues encompassed in a DNA binding region predicted by PreDs with a Parea of greater than 250 Å^2^ was specified as active site residues whereas passive residues were automatically defined around the active site which forms the boundary of the DNA-binding region. This specification was introduced to enhance the conformational search space for docking simulation as well as to avoid blindfold docking experiments. Definition of residues takes the form of experimental data which were converted into ambiguous interaction restraints (AIRs) in order to generate topology of the structures subsequently. The docking procedure consists of three stages: an energy minimization in a rigid-body manner, a semiflexible refinement in torsional space, and a final refinement in explicit solvent. After execution of each of these stages, the resultant structures are scored and ranked and the best fitted structures are employed in next stages. The best docked conformation can be obtained (usually clustered at the top) by inspecting the HADDOCK score which is a summation of intermolecular energies, namely, van der Waals (vdW), electrostatic (Elec), desolvation (Dsolv) and AIRS together with buried surface area (BSA): rigid-body score = 1.0 ∗ Elec + 1.0 ∗ vdW−0.05 ∗ BSA + 1.0 ∗ Dsolv + 1.0 ∗ AIR; final score = 1.0 ∗ Elec + 1.0 ∗ vdW + 1.0 ∗ Dsolv + 1.0 ∗ AIR.

### 2.12. Generation of Electrostatic Potential Map for Docked Structures

The influence of electrostatics for enabling DNA-protein interaction was studied using continuum Poisson-Boltzmann (PB) electrostatic approach. It was achieved by PBEQ-Solver (PBEQuation-Solver; http://www.charmm-gui.org/?doc=input/pbeqsolver) [39] for which PQR files were required as molecular inputs. Hence, the docked complexes in PDB format were converted into PQR format using PDB2PQR server (http://nbcr-222.ucsd.edu/pdb2pqr_1.8/) [40]. PQR format embodies the replacement of occupancy column in a PDB file (“*P*”) with the atomic charge (“*Q*”) and the temperature factor column with the atomic radius (“*R*”). The inputted PDB file was subjected to following structural manipulations: rebuilding missing heavy atoms, building and optimizing hydrogens and assignment of atomic charges and radii based on force field parameters from CHARMM22 (selected option), AMBER99 or PARSE. All the PB calculations on PBEQ-Solver were performed in a coarse grid space (before focusing = 1.5 Å and after focusing = 1.0 Å) and utilized molecular surface (computed with a probe radius of 1.4 Å) to set up the dielectric boundary. The resultant electrostatic potential grid map in data explorer (*dx*) format was recovered and specified as input to PyMol version 2.5 program (academic version; Schrodinger LLC) [41] to view the PBEQ electrostatic map.

## 3. Results and Discussion

### 3.1. Prediction of Protein Family of Coat Protein

**Figure 1:**
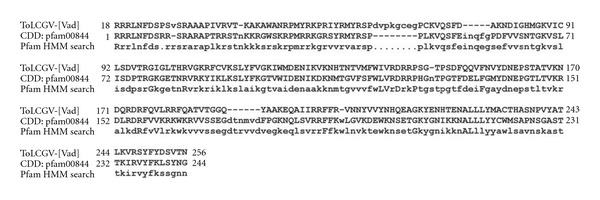
Sequence alignment of ToLCGV-[Vad] coat protein with nuclear export factor of BR1 family (Pfam entry: 00844 recovered from NCBI CDD) and HMM profile of the geminivirus coat protein.

The protein family of ToLCV coat protein was predicted using a combination of programs. NCBI CD-Search using protein sequence revealed that it belongs to Gemini-coat protein superfamily (Pfam entry: pfam00844, accession no. Q8QYY9). Upon carefully examining the sequence alignment generated (*E*-value: 5.53e-100; bit score: 290.36) with SqLCV BR1 nuclear shuttle protein, it was studied that ToLCGV belongs to nuclear export factor BR1 family (Figure 1). BR1 is a ssDNA binding protein that shuttles between the nucleus and cytoplasm in plant cells [33].

PSI-BLAST sequence hit (PSI-BLAST threshold: 0.005 maximum iterations: 7; *E*-value: 1e-105; bit score: 383; sequence coverage in alignment: 99.64%) with a capsid protein of *Begomovirus* taxa (UniRef90 P03560; tomato golden mosaic virus) was observed. Further, sequence-based query over Pfam-A (Pfam-B not chosen as we focused on obtaining highly curated data) database produced a result similar to NCBI CD-Search. This HMM-based search provided an alignment with an *E*-value of 2.3e-87 and bit score of 292.1 (Figure 1). Manual inspection of PubMed references in the pfam00844 entry in NCBI CDD disclosed that coat proteins of *Geminiviridae* family binds ss- and ds-viral DNA *in vitro *[42]. For instance, TYLCV coat protein [43], maize streak virus (MSV) coat protein [21], SqLCV nuclear shuttle protein [44], and bean dwarf mosaic geminivirus(BDMV) movement protein [34] have the same function of binding which helps them to establish infection by nuclear shuttling of viral DNA across cell boundaries. Besides, coat protein also possesses binding function necessary for encapsidation of viral DNA. It is well known that the genomic component DNA-B in bipartite *Begomovirus* such as ToLCNDV encodes two movement proteins, namely, nuclear shuttle protein (NSP) and cell-to-cell movement protein (MP) that direct the viral genome to the cortical cytoplasm and across the barrier of the cell wall for infection multiplication [45]. On the other hand, monopartite *Begomovirus* including ToLCGV [9] produces coat protein and other associated proteins to alleviate movement inside the host while coat protein acts as nuclear shuttler facilitating import and export of DNA [46]. Therefore, it is anticipated that coat protein of ToLCGV may also function as nuclear shuttler.

### 3.2. Prediction of NLS in the Coat Protein Sequence

Mutagenesis study on MSV coat protein [4] and TYLCV [5] NLS region resulted in the cytoplasmic accumulation of the mutant protein. Thus, ToLCV coat protein must possess a NLS region in its sequence in order to be translocated to nucleus. A NLS signal was predicted with a score 10.2 by the cNLS Mapper in the coat protein N-terminal with a composition of 20 amino acids (predicted bipartite NLS: MSKRPADMLIFTPASKVRRR, predicted monopartite NLS: none).

**Figure 2:**
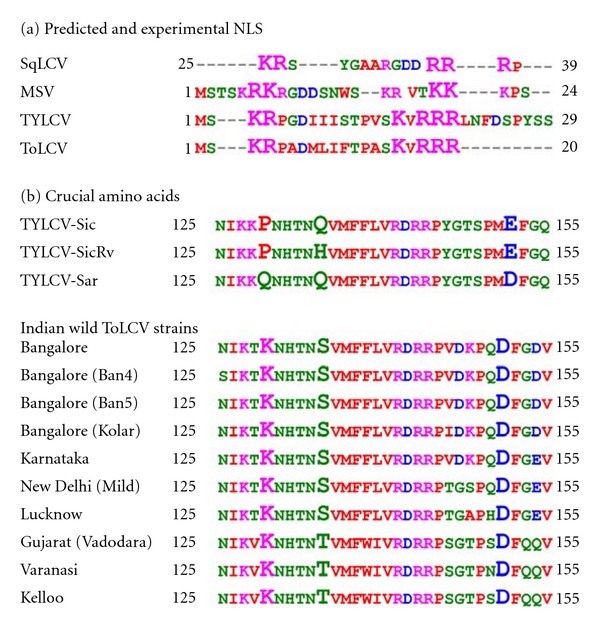
(a) MSA of predicted and experimental NLS of selected geminivirus coat proteins. (b) MSA of coat proteins from Indian and non-Indian strains corresponding to sequence region of interest containing the key amino acids (highlighted in larger fonts) required for systemic infection, viral particle formation, and insect transmission.

The occurrence of basic amino acids in the predicted NLS showed that lysine and arginine constituted 2 and 4 counts which proposed to have a classic NLS pattern (Table 1) and can be comparable to experimentally identified TYLCV coat protein NLS [5] (Figure 2(a)). Despite the impressive number of receptor-cargo interactions that have been studied, the prediction of NLSs in candidate proteins remains extremely difficult. So, we step forwarded our search in scientific literatures related to BR1 nuclear export family in order to infer the predictions made. Pairwise sequence alignment of NLS region from MSV and ToLCGV-[Vad] coat proteins resulted in an identity and similarity percentage of 25% and 33.3% with a score of −6 whereas TYLCV and ToLCGV-[Vad] yielded an identity and similarity percentage of 51.7% and 58.6% with a score of 49. This pairwise alignment suggested that ToLCV coat protein is much more conserved with TYLCV rather than SqLCV [3] (identity and similarity = 15%, score = −20), a representative protein member of BR1 family (pfam00844) in Pfam database.

### 3.3. MSA and Phylogenetic Analysis of Indian Strains

ToLCV coat protein sequences from Indian strains were retrieved from NCBI database to construct MSA in order to study amino acids crucial in conserved domain and responsible for systemic infection, viral particle formation, and insect transmission. Norris et al. 1998 reported that a functional coat protein having amino acids in the following sequence positions, namely, Pro/Gln129, Gln/His134, and Glu/Asp152 on TYLCV isolates is essential for correct assembly of virions and transmission by the insect vector [47]. These key residues were identified by *B. tabaci* transmissibility studies in the field isolates of TYLCV-Sic (Sicily), TYLCV-Sar (Sardinia), and TYLCV-SicRv (engineered mutant of Sicily) [47]. Examination of corresponding positions in our MSA cluster revealed that Lys129, Ser/Thr134, and Asp151 (instead of 152nd position as a result of single residue deletion) were conserved amongst Indian strains in comparison to non-Indian isolates and are found to be wild-type. The comparison of chemical properties of the template with that of MSA showed that a positively charged amino acid (Lys129) was identified in the uncharged polar (Gln129) position. The second important residue (Ser/Thr134 in replacement with Gln/His134) was conserved in terms of polarity while a negatively charged residue (Glu/Asp152; Asp151) was preserved in the third crucial position (Figure 2(b)). This amino acids combination (Lys129, Ser/Thr134, and Asp121) is also conserved in coat proteins among different wild-type viruses, namely, tomato golden mosaic virus, tomato mottle virus-[Florida], sinaloatomato leaf curl virus, tomato leaf crumple virus, taino tomato mottle virus, abutilon mosaic virus-[Hawaii], bean golden mosaic virus-[Brazil], SqLCV and papaya leaf curl virus [47].

**Figure 3:**
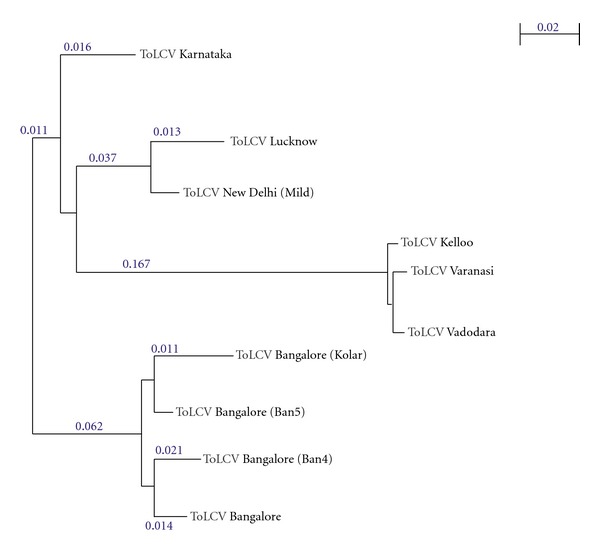
Phylogenetic tree of ToLCV coat protein representing closeness among Indian strains.

A phylogenetic tree based on NJ algorithm was constructed using Phylip version 3.68 to study the sequence conservativeness among Indian strains. Surprisingly, coat proteins characteristic from districts, namely, Vadodara, Varanasi, and Kelloo were clustered in a node with a branch length of 0.167. It should be noted that these members were representing different states in the Northern India contrasting to other members which were sufficiently diverged to each other. Besides the fact that Bangalore isolates were conserved among each other, they were distinct with one of the state member, Karnataka with a length of 0.011. Isolates from New Delhi and Lucknow were conserved as expected in terms of area nearness (Figure 3). The key amino acids required for biochemical functions were indeed conserved amongst each other with respect to the comparison using MSA made above.

### 3.4. Disorderness and Their Link with Predicted NLS

**Figure 4:**
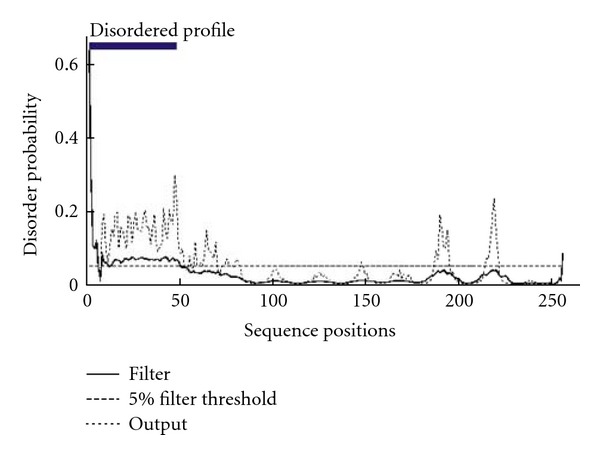
Disordered profile of ToLCV coat protein with disorderness in the sequence positions 1–50 which contains predicted NLS.

Disorderness was predicted in the coat protein to identify the sequence regions that cannot be modeled efficiently with the protein modeling procedures adopted by us. This scrutiny was taken to eliminate the loop region in the sequence terminals. Disorder profile produced with 5% filter threshold showed that a window with sequence positions from 1 to 50 was scattered with peaks demonstrating residue disorderness (Figure 4). This region corresponds to NLS in the N-terminal. There exists a relationship between the predicted NLS and the disorderness as the corresponding sequence position was predicted as loop region with a variety of secondary structure prediction programs including PSI-Pred, GORIV, and so forth. So, we decided to exclude NLS signal from the protein sequence for modeling due to the consideration of disordered profile and the increased possibility of generating loop geometry.

### 3.5. Structure Modeling of Coat Protein

No close template was obtained in an attempt to find structurally known homolog of ToLCV coat protein using BLASTp and sequence based BLAST search in PDB with default settings. Therefore, we decided to use homology domain modeling using Robetta program and remote-based homology modeling using Phyre program.

After evaluation of predictions related to secondary structure features, Ginzu, a domain parser of Robetta modeled two domains with a sequence span of 1–59 and 60–235 using Pfam and HHSearch as sources. K∗Sync alignment method was employed subsequently to generate structurally good scoring decoys followed by loop modeling, domain assembly, and side-chain packing. This procedure yielded five models. Remote-based homology modeling using Phyre provided a model based on the Nucleoplasmin-like/viral protein (viral coat and capsid proteins) as folding unit (PDB ID: 2BUK chain A; structure of satellite tobacco necrosis virus after crystallographic refinement at 2.5 Å resolution) [48] derived from satellite virus with a confidence of 97.3. Fortunately, Robetta used the same structural template for models generation. Phyre provided a list of other models sorted by its confidence level were found to only range from 8.03 to 23.9.

### 3.6. Selection of Best Scoring Models

**Figure 5:**
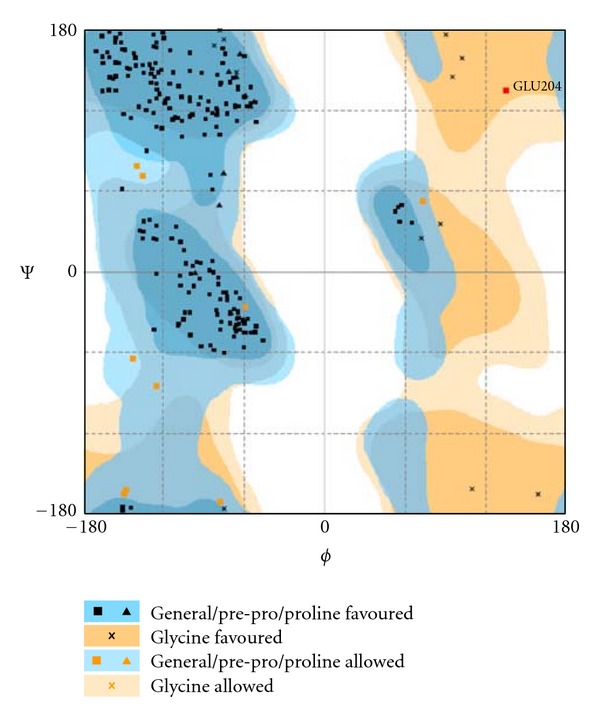
Best scored ToLCV Robetta model showing one outlier (Glu204) on Ramachandran plot.

A total of six coat protein models (five obtained from Robetta program and one from Phyre program) were subjected to energy minimization with AMBER7 force field and 250 as maximum number of evaluations using minimize energy module engineered in Tripos Benchware 3D Explorer. Energy minimized structures were then validated using stereochemistry check with the help of Ramachandran plot. The Robetta model (energy minimized to −2682.00 Kcal/mol) was chosen based on the residue occurrence of more than 95% estimated by summing up favorable and allowed regions (Figure 5) in *φ*-*ψ* core areas and the presence of only one outlier (Glu204) whereas the best scored Phyre model was discarded due to the loose packing of loop regions and its close resemblance to Robetta model (root mean squared deviation (RMSD): 20.8578 Å over 1041 matched atoms). There is one more reason to unconsider the Phyre model as the N-terminus was constituted with loop elements, two helices in the intervening region (two helices expected at the N-terminal) and eleven *β*-strands (only eight were expected instead of eleven) as it was not complied with characteristic Geminate viral particle. So, we discarded these models for further analysis.

### 3.7. Resemblance to Geminate Viral Particle

**Figure 6:**
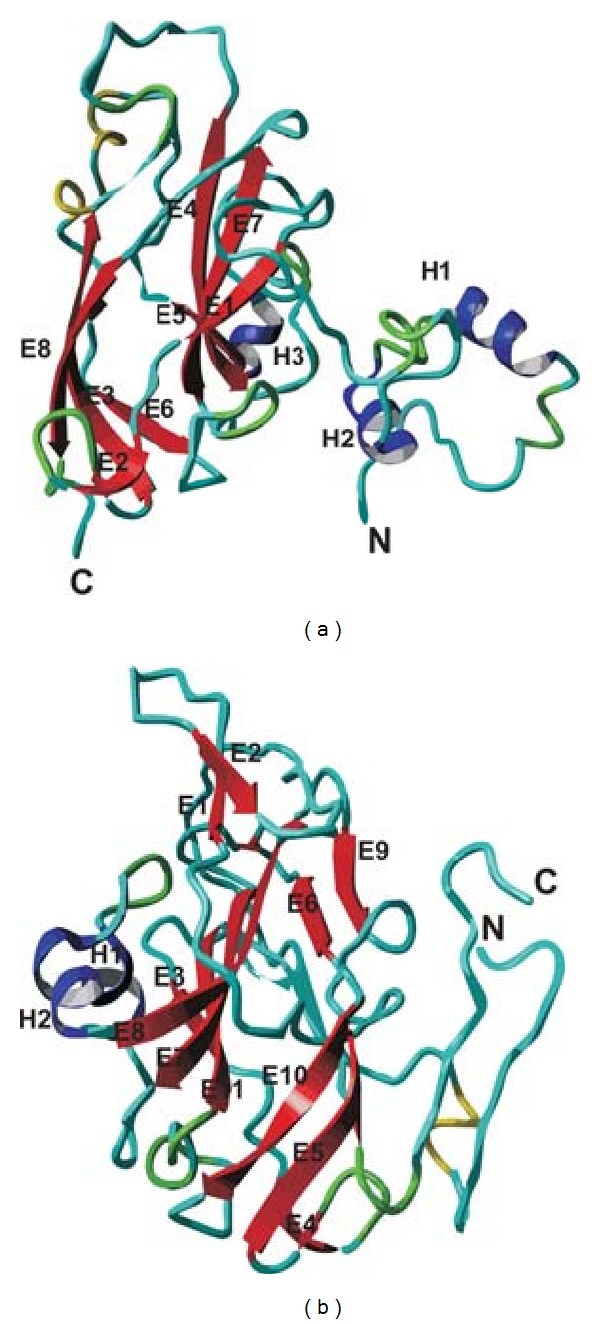
Developed ToLCV models using homology domain modeling (Robetta program; (a)) and remote-based homology modeling (Phyre program; (b)). H-Helix, E-Extended *β*-strands.

The modeled ToLCV coat protein was compared with the structure of the MSV Geminate particle, a member of Geminiviridae family determined using cryo-electron microscopy and three-dimensional image reconstruction methods [49]. The modeled protein possessed an N-terminal helix with an eight-stranded antiparallel *β*-barrel motif characteristic of Geminate particle. The *β*-barrel motif is a dominant structural unit in all ssDNA virus structures that have been determined to atomic resolution. Unlike the model generated by Zhang et al. 2001 [49], our Robetta model has 7 reliable and 1 short (total 8 strands) antiparallel *β*-strands and 2 helices at the N-terminal instead of 1 in comparison to Zhang's Geminate model. We also noticed 1 helix at the intervening region (Figure 6). We expected that this additional accumulation of secondary structural elements is beyond the evolution and may be an additional procurement in the dsDNA virus structures in disparity to ssDNA virus structures due to conservational pressures as described by Zhang et al. 2001 [49] or due to the insertional sequences or it may be due to the loop geometry as it was undistinguished by the present programs due to the nonavailability of experimental atomic information.

### 3.8. Sequence and Structure-Based DNA Binding Properties

**Figure 7:**
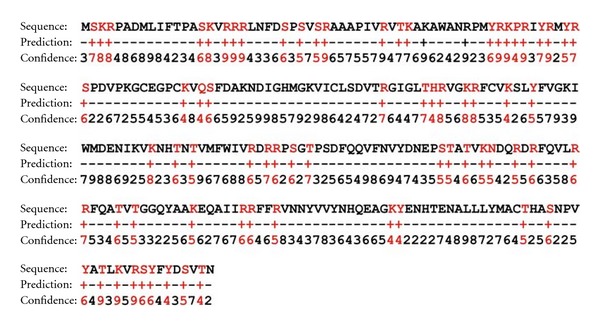
Sequence-based DNA binding properties of ToLCV coat protein. DNA binding residues are shown in red text.

**Figure 8:**
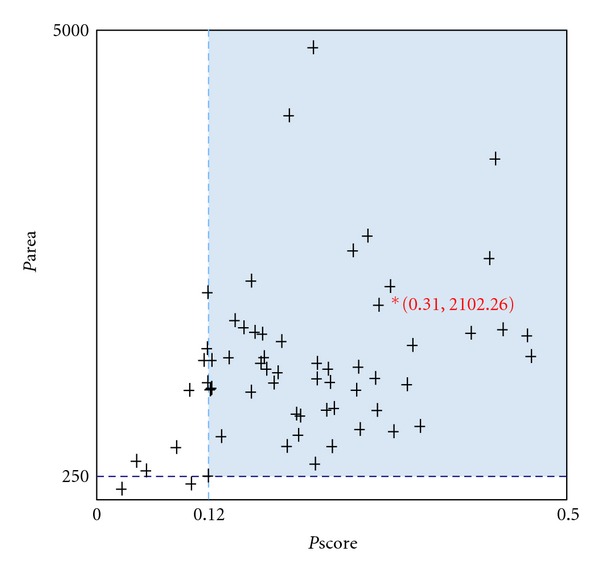
Structure based DNA binding properties of ToLCV coat protein having reliable Pscore and Parea.

ToLCV must possess DNA binding properties for accomplishing several cellular functions such as nuclear targeting of viral DNA, encapsidation of viral DNA, systemic infection, viral particle formation, insect transmission, and correct assembly of virions as experimentally studied in other members of the family, namely, MSV, SqLCV, BDMV, and so forth. Interestingly, the coat proteins bind both ss- and ds-viral DNA in sequence independent fashion [21, 33, 34]. To reveal the crucial amino acids involved in DNA interaction, sequence- and structure-based approaches were utilized. BindN predicted spatially distributed residues as component of DNA binding interface with 74 amino acids and 28.90% contribution (Figure 7). We expected that this widespread distribution of DNA binding residues will come together during protein folding and will interact with viral DNA. Thus, we step forwarded to identify those prominent amino acids using our generated ToLCV coat model. PreDs revealed loop regions as potential DNA binding region accompanied with all those amino acids predicted by BindN. This prediction was validated by inspecting the scoring functions such as Pscore and Parea. Pscore is an indicator for the ratio of the predicted area possessing maximum value while Parea represents area of the predicted ds-DNA binding region on the protein surface. We achieved 0.31 as Pscore (threshold: >0.12) and  2102.26 Å^2^ (threshold: >250 Å^2^) as Parea, respectively (Figure 8).

The TYLCV coat protein gets imported into the plant and insect cells nuclei via using its N- terminal NLS [5]. It is also been proposed that TYLCV coat protein functions as BR1 protein facilitating DNA trafficking across cell boundaries and demonstrated that the coat protein binds DNA cooperatively [43]. It is also highlighted that TYLCV coat protein may also aid in protecting the transported coat protein-DNA complex from intracellular nucleolytic degradation as this complex was highly resistant to *in vitro* S1 nuclease activity [43]. In MSV coat protein, the DNA binding domain was mapped to the N-terminal 104 amino acids inclusive of NLS [21]. Immuno-electron microscopy revealed that DNA binding domain between residues 5 and 22 suggested that this region could be involved in transporting geminivirus coat protein towards nuclei [50]. We predicted that certain N-terminal amino acids of ToLCV coat protein scored a confidence of 6–9 indicating DNA binding properties (Figure 7). Besides, structural analysis of modeled ToLCV indicated that N-terminal residues contributing to loop secondary structure form the major element in interacting with viral DNA as described below.

### 3.9. Viral DNA Structure Modeling and Docking with Coat Protein

**Figure 9:**
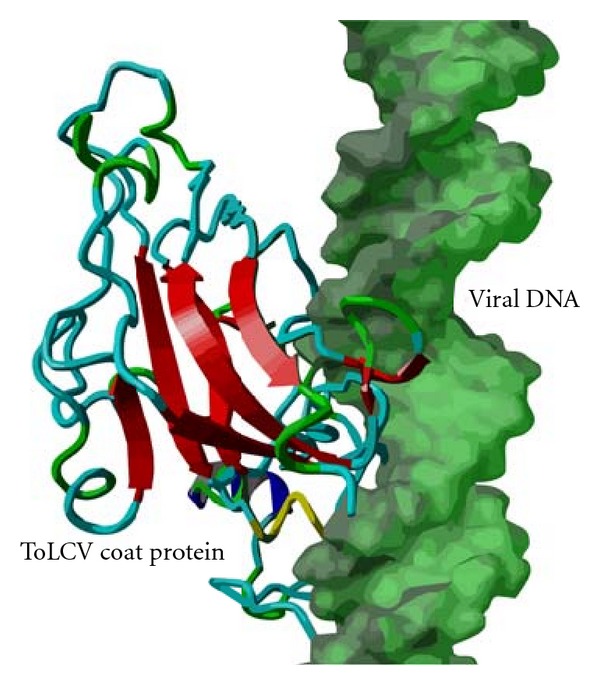
The binding mode of ToLCV coat protein with viral dsDNA where loop element of protein and major groove of DNA acting as interfaces.

We developed a canonical ds-viral DNA using 3D-DART with generalized geometrical constraints to explore the interaction with coat protein. Molecular docking was performed using HADDOCK program with predicted DNA binding region as active site. Best scoring clusters were sorted based on HADDOCK score. The most reliable (top) cluster having four similar docked conformations (HADDOCK score: −12.0 ± 11.0) were recovered. The DNA binding interface of coat protein was found to be the loop region whereas major groove was the molecular interface of viral DNA in which the best conformers were sampled (Figure 9). The intermolecular energies (unit in KJ/mol) obtained are as follows: vdW: −66.9 ± 5.5, Elec: 827.4 ± 87.8, Dsolv: 125.7 ± 11.7, AIR: 947.0 ± 50.09, and BSA: 2099.6 ± 137.9. We also observed that RMSD of overall lowest-energy structure was 4.3 ± 3.0 with respect to structures in different clusters. The internal energy in apo form (free molecules) was 278722.00 KJ/mol whereas in bound form (DNA-protein complex) was 15494.00 KJ/mol and the binding energy was predicted as −264139.00 KJ/mol.

### 3.10. Electrostatic Interaction of Coat Protein

**Figure 10:**
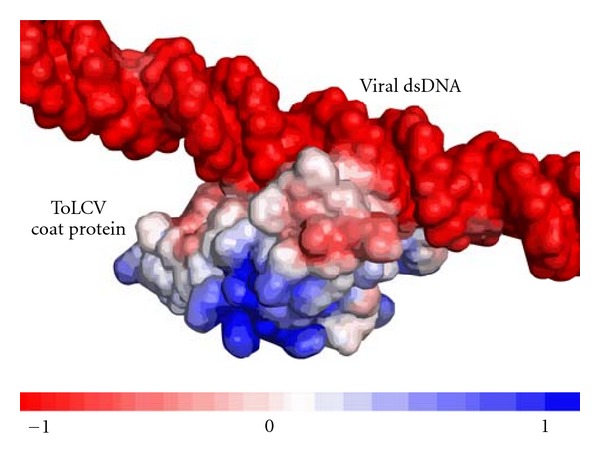
Electrostatic potential map of ToLCV coat protein-viral dsDNA complex. Neutral patch of coat protein interacts with negatively charged DNA.

DNA binding interface predicted by PreDs was found to be electrostatically favored as its prediction was principally based on electrostatic potential, local, and global curvatures present on the DNA surface. HADDOCK revealed coat protein's loop topology as its molecular interface unit. DNA-protein interaction is predominantly influenced by electrostatics and can be efficiently studied using adaptive Poisson-Boltzmann solver (APBS) approach. The docked conformation was charged appropriately based on physiological environment with preprocessing using PDB2PQR and all PB calculations were carried out using PBEQ-Solver in a coarse grid space. Electrostatic grid map analyzed using PyMol showed that neutral patches in coat protein were found to interact with viral DNA (Figure 10). We expected that an isosurface of positive patch will tend to interact with negatively charged viral DNA. Upon manual inspection of charged clusters in the coat protein, the positive charged clusters were cornered in surface with significantly low negative patches and a greater deal of positive regions corresponded to *β*-barrel motif. We ruled out the requirement of positive patch contributing to DNA interaction as geometrical flexibility of loop region in the coat protein having neutrality was much favored rather than charge-charge attraction. This view was further inferred by the best docked conformation in which electrostatic energy term was investigated and found to be the major intermolecular descriptor representing interaction.

Zrachya et al. 2007 reported that TYLCV coat protein could be targeted by small interfering ds RNAs (siRNAs) derived from intron-hairpin RNA construct to develop disease resistant transgenic tomato cultivars and showed its potential in *N. benthamiana* transient assays [51]. Similar studies targeting against antisense replicase gene (AC1) in ToLCV helped in developing trait-stable transgenics [52]. Besides, the midsized aggregation of coat protein inside nucleus is associated with resistance whereas large aggregates leading to infection susceptibility [53]. We highlighted sequence regions in ToLCV coat protein possessing both DNA binding properties and the functional amino acids combination essential for virulence and these regions of interest can be targeted by developing siRNA. In addition, these molecular properties of ToLCV coat protein can be accounted in developing *Begomovirus* resistant-engineered plants.

## 4. Conclusion

ToLCV coat protein possesses DNA binding properties to function similar to BR1 nuclear shuttler. The amino acid combinations crucial for virulence were investigated through MSA and the evolutionary relationship was traced by phylogenetic analysis which indicated that the Indian strains are closely related in the context of geographical locations. The predicted NLS of ToLCV coat protein shares more similarity with experimentally known TYLCV coat protein NLS. Molecular modeling represented ToLCV coat protein as Geminate viral particle. Further, sequence- and structure-based approaches identified that ToLCV coat protein through its loop topology interacts with viral DNA as surface complementarity proven to be the major promoting factor followed by electrostatic interaction. We anticipate the conserved region of ToLCV coat protein prominent for DNA binding and functional sequence pattern can be targeted by RNA interference to develop disease-resistant transgenic tomato plants.

## Figures and Tables

**Table 1:** Known and predicted NLS pattern of BR1 nuclear export family.

Organism	Predicted NLS pattern	Frequency of lysine residues	Frequency of arginine residues
SqLCV coat protein	KRSYGAARGDDRRRP (Sanderfoot et al., 1996 [3])	1	5
MSV coat protein	MSTSKRKRGDDSNWSKRVTKKKPS (Liu et al., 1999 [4])	6	3
TYLCV coat protein	MSKRPGDIIISTPVSKVRRRLNFDSPYSS (Kunik et al., 1998 [5])	2	4
ToLCGV-[Vad]	MSKRPADMLIFTPASKVRRR (predicted by cNLS Mapper)	2	4
